# Capillary haemangioma of the heart presenting with pericardial effusion: A case report

**DOI:** 10.34172/jcvtr.2020.60

**Published:** 2020-12-23

**Authors:** Anshuman Darbari, Devender Singh, Shegu Gilbert, Barun Kumar, Neha Singh

**Affiliations:** ^1^Department of CTVS, AIIMS, Rishikesh-249203, India; ^2^Department of CTVS, KMCH, Coimbatore-641014, India; ^3^Department of Cardiology, AIIMS, Rishikesh-249203, India; ^4^Department of Pathology, AIIMS, Rishikesh-249203, India

**Keywords:** Cardiac Tumours, Capillary Haemangioma, Pericardial Effusion

## Abstract

Cardiac haemangiomas (CH) are rare benign primary tumours of the heart and constitute nearly 2.8% of primary cardiac tumours. In a-48-year-old female, a cardiac tumour mass over right ventricular out flow area and main pulmonary artery was detected during diagnostic workup for aetiology of recurrent pericardial effusion. Echocardiograhy and pericardial fluid findings were non conclusive. Contrast enhanced Computed tomography (CECT) and Positron emission tomography (PET) scan imaging found the exophytic, moderately hypermetabolic, heterogeneous mass lesion posterolateral to main pulmonary trunk. We did partial resection of lesion without cardiac reconstruction and open incisional biopsy through midline sternotomy incision. Histopathological analysis confirmed this as a case of Capillary type of haemangioma of heart.

## Introduction

CH is a rare clinical entity. Most cardiac haemangiomas are asymptomatic, although various symptom association are described occur depending on the size and location of the tumour. CH can arise from any layer of the heart and in any of the cardiac chambers. The right atrium is the predominant location followed by the left ventricle for CH.^1,2^ The natural history of CH is still unknown. We found comprehensive case analysis of CH based on literature review article by Weidong Li et al. The clinical demography, clinical features, histopathologic features, treatment and prognosis of these cases are analysed but they also highlighted their study limitations due to rarity of these cases and non-availability of randomized, controlled studies.^3^ Now PET, Computed tomography (CT) and Magnetic resonance imaging (MRI) have proven very useful for preoperative diagnosis of cardiac tumours. This case report will also add in data base and moreover recurrent pericardial effusion due to cardiac capillary haemangioma is not described earlier.

## Case Presentation

**Figure 1:**
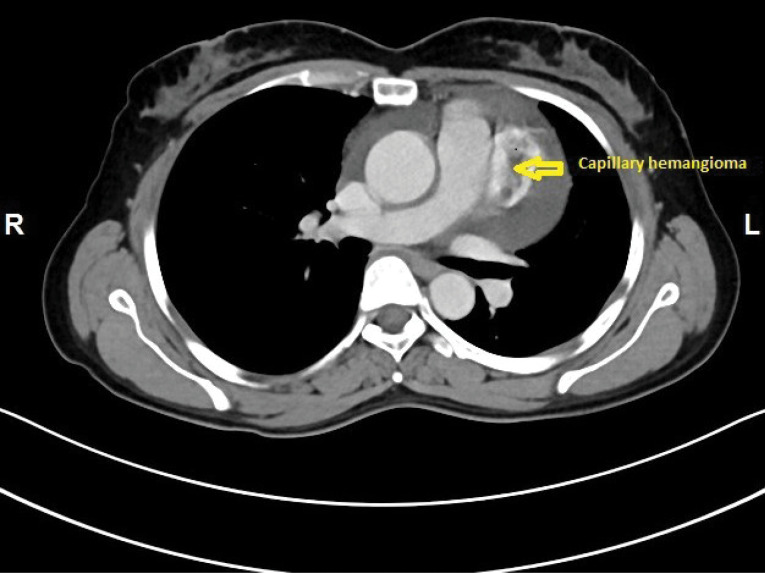
Contrast enhanced computed tomography axial plane view showing a relatively heterogeneous capillary haemangioma mass (48 × 30 mm) marked by arrow, posterolateral to main pulmonary artery infundibulum.

**Figure 2:**
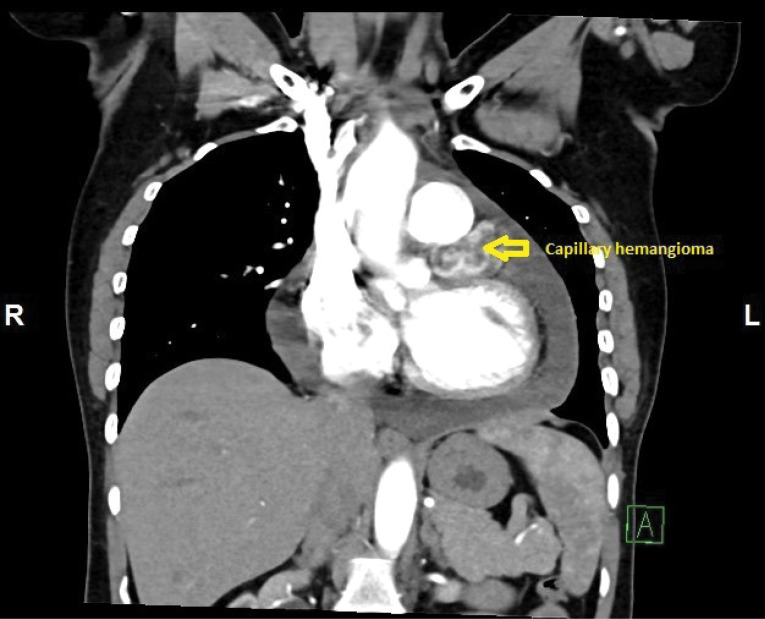
Contrast enhanced computed tomography coronal plane view showing a relatively heterogeneous mass (48 × 30 mm) marked by arrow, Superio-anterior to Left ventricle.

A 48-year-old female was admitted with history of breathlessness since 3 months. On exploring clinical history, it was found that she also had recurrent history of giddiness on and off with mild chest discomfort since 6 months. She was non diabetic and normotensive. General clinical examination and cardiovascular examination was unremarkable. Initial 2-D Transthoracic Echocardiography revealed large pericardial effusion with mild Pulmonary arterial hypertension without any other valvular, cardiac chamber or flow abnormality. She underwent echo guided pericardiocentesis for diagnostic and therapeutic purposes and initially 650 ml of straw coloured, slightly turbid fluid was drained. The pericardial fluid examination revealed exudative fluid but Lactate dehydrogenase (LDH), Adenosine Deaminase (ADA) test reports were within normal limits. Gram staining and Ziehl-Neelsen staining for Acid fast bacilli were negative. The cytological examination of fluid showed mesothelial cells and inflammatory cells in haemorrhagic background but without any malignant cells. Antinuclear antibodies and Anti-double stranded DNA antibodies were also found to be negative. Haematological and routine biochemical tests are also within normal limits. Since all the tests were negative she was labelled as idiopathic pericardial effusion and kept under medical supervision with diuretics and low dose steroids. After one month, she again developed moderate pericardial effusion with recurrence of symptoms. She was planned for Pericardial window formation. We started further investigations. CECT thorax revealed well defined middle mediastinal mass of 43 x30 mm superior to left ventricle and posterolateral to main pulmonary artery infundibulum without any compression (Figure 1 and 2). PET scan showed, heterogeneous enhancing exophytic, moderately hypermetabolic heterogeneous mass lesion posterolateral to main pulmonary trunk and anterior to the left ventricle with SUV Max of 4.0 and no other evidence of hypermetabolic lesion elsewhere in thoracic cavity.

**Figure 3:**
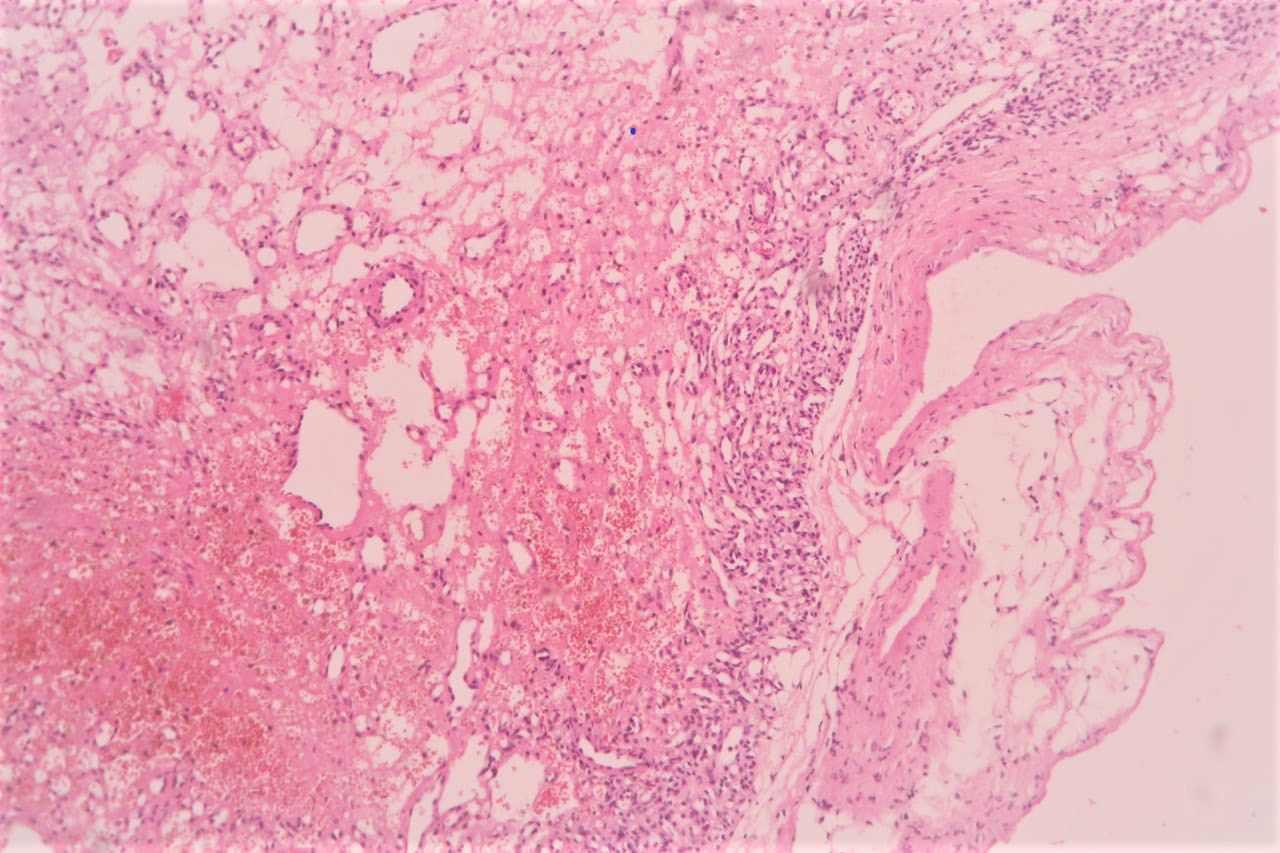
Microscopic view of section shows an encapsulated tumour composed of numerous small capillary sized vascular spaces, some of which are dilated. These spaces are lined by endothelial cells. Stroma shows areas of oedema and haemorrhage. (x100, Hematoxylin & Eosin).

**Figure 4:**
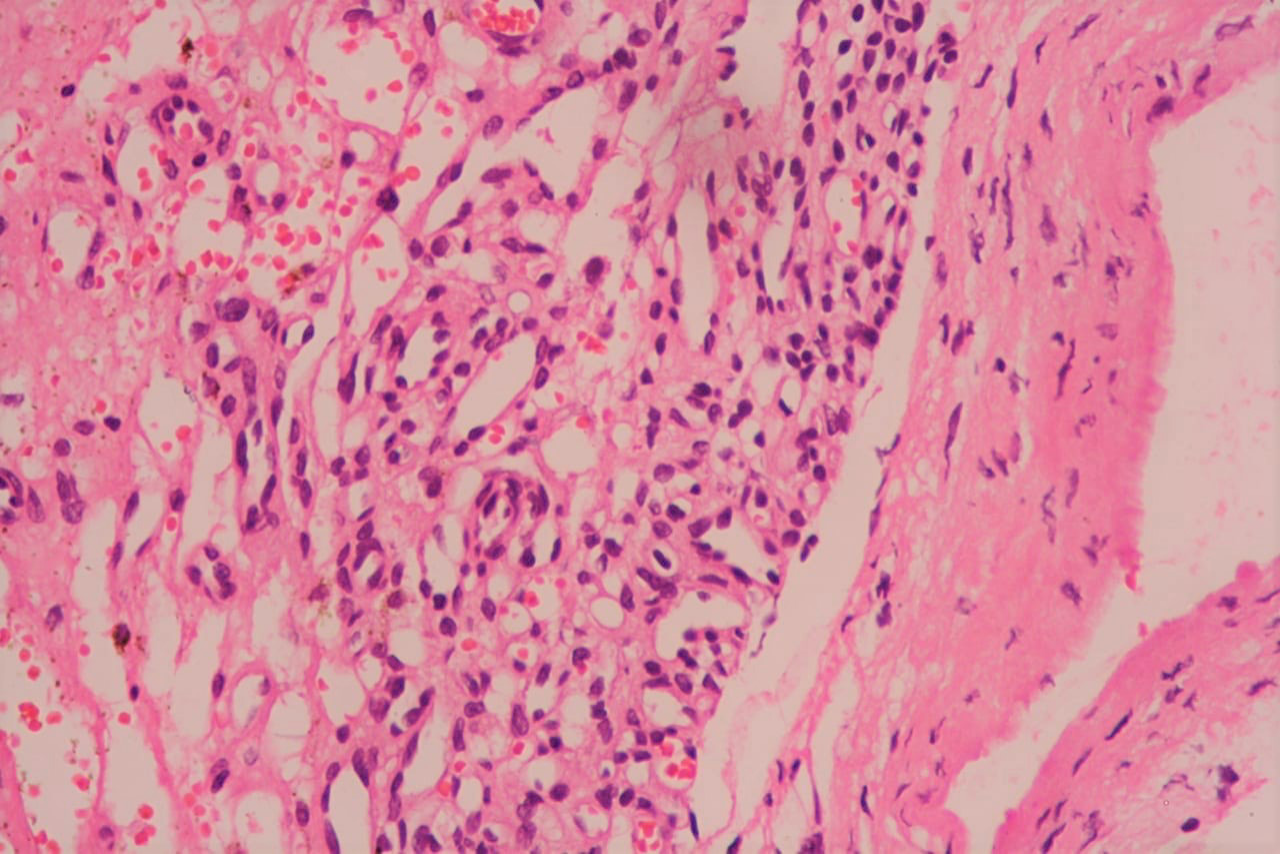
High power microscopic view showing tumour composed of tiny capillary sized vascular spaces which are lined by plump endothelial cells. Scant fibrous stroma with foci of haemorrhage are also seen. (x400, Hematoxylin & Eosin).

The patient underwent midline sternotomy under general anaesthesia for planned pericardial window surgery with diagnostic and therapeutic intent for the mass after thorough preoperative workup including ultrasonography of Abdomen and pelvis region. After opening pericardium, we found encapsulated, firm, highly vascular mass immediately adjacent to the main pulmonary artery and extending towards the posterior wall of right ventricle. Since the mass seems to be part of the right ventricular wall, resectability was deemed as difficult. Hence, only careful partial mass resection without any cardiac reconstruction was done after taking pledgeted polyprolene sutures around planned excision areas, to prevent torrential bleeding or cardiac rupture. Overlying total pericardium was removed to make left sided pleuropericardial window. Procedure went smoothly and post operatively patient did well. The histopathology examination revealed multiple vascular like structures and small capillary type vessels suggestive of a capillary type haemangioma without mitosis (Figure 3 and 4). She is currently asymptomatic with no recurrence of any pericardial or pleural effusion after regular follow-up of six months.

## Discussion

The approximate overall incidence of primary cardiac tumours is reported seventeen in a million at autopsy. CH constitutes represents only 2.8% of primary cardiac tumours. The natural history of cardiac haemangiomas is unclear because they are most often reported as isolated case report or found incidentally on autopsy.^1,2^ This rare, benign tumour occur sporadically most commonly in the fifth decade of life. The clinical presentation varies and depends on location, size, rate of growth and distinct. Majority of cases reported are usually asymptomatic until hemodynamic changes and local invasion lead to other complications. CH can arise from any layer of the heart (endocardium, myocardium, epicardium and pericardium) and develop in any of the cardiac chambers. There are case reports of CH invading the conductive tissue or septum (interatrial or interventricular) of heart. Despite being benign in nature, these patients are under higher risk of CH-related sudden preoperative death. So an active attitude towards treatment of the CH is required in these locations despite its histopathologic benignity due to unusual risk of life-threatening complications like syncope, stroke, and even sudden death.^3^

Mostly it is a solitary tumour with lack the ability to metastasize and very slow growth rate. These tumours are well circumscribed by an outer surface. Pathologically, CH are characterized by benign proliferative endothelial cells lining blood vessels with increasing vascularization and focal tuft formation. Mitoses are rarely found. Electron microscopy shows interdigitating endothelial cell borders without desmosomes or tight junctions. Histopathologic features of CH are same to those of haemangiomas elsewhere in the body. Based on the predominant type of the proliferating vessels, CH are classified histologically into the three types: capillary type with small capillary-like vessels, cavernous type with multiple dilated thin walled vessels and arterio-venous type with dysplastic arteries and veins. As, most haemangiomas exhibit overlapping features with interspersed fibrous tissue it is also frequently difficult to distinguish them from other types of malignant tumours.^3^

The routine chest roentgenogram and Electrocardiography (ECG) tests are nonspecific for diagnosis. Echocardiography is the most important diagnostic tool because of easy availability, non-invasiveness. In addition, echocardiography enables high accuracy real-time observation, especially with intracardiac lesions. Advanced imaging studies as CT, MRI and PET now offer preoperative diagnosis with exact anatomical structural relations and these investigations offer the advantage of differentiating CH from metastatic lesions. But, the histopathologic diagnosis is the gold standard investigation.^3,4^

Based on the low incidence of postoperative long-term adverse events and recurrence in the case series analysis, Weidong et al suggested complete surgical excision as the first-line therapy. Radical excessive tumour removal deemed by them unnecessary because partial tumour resection is found equally effective as complete removal. Biopsy alone is not suggested by them owing to its high incidence of long-term adverse events. Corticosteroid therapy, radiotherapy, vascular endothelial growth factor antagonists, and b-receptor blockers are also reported and tried especially in patients with unresectable CH without sufficiently evidence-based controlled studies.^3^ Prognosis is favourable for most patients with haemangiomas, it is important to evaluate the characteristics of the tumour with the precise accuracy because cardiac reconstruction, which may require after wide excision may presents a technically complex challenge.^4,5^ There are very rare reports of pericardial effusion and hemopericardium due to this pathology as in our case.^7,8^ Partial resection with biopsy of lesion confirmed the diagnosis of Capillary type of CH.

## Conclusion

CH are rare benign cardiac tumours that occur most frequently in the right side of the heart as in our case. Echocardiography, CECT Scan and PET scan imaging studies are required before any operative plan. Histopathological diagnosis is gold standard for confirmation. Currently, the recommendations for management of CH are evolving because of rarity of cases and absence of randomized, controlled studies.

## Competing interests

None.

## Ethical approval

The authors certify that they have obtained all appropriate patient consent forms. In the form the patient(s) has/ have given his/her/their consent for his/her/their images and other clinical information to be reported in the journal. The patients understand that their names and initials will not be published and due efforts will be made to conceal their identity, but anonymity cannot be guaranteed.
